# Simultaneous Use of ROCK Inhibitors and EP2 Agonists Induces Unexpected Effects on Adipogenesis and the Physical Properties of 3T3-L1 Preadipocytes

**DOI:** 10.3390/ijms22094648

**Published:** 2021-04-28

**Authors:** Yosuke Ida, Megumi Watanabe, Hiroshi Ohguro, Fumihito Hikage

**Affiliations:** Departments of Ophthalmology, Sapporo Medical University School of Medicine, S1 W17, Chuo-ku, Sapporo 060-8556, Japan; funky.sonic@gmail.com (Y.I.); watanabe@sapmed.ac.jp (M.W.); ooguro@sapmed.ac.jp (H.O.)

**Keywords:** ROCK inhibitor, EP2 agonist, 3-dimension tissue cultures, 3T3-L1 cell

## Abstract

To elucidate the additive effects of an EP2 agonist, omidenepag (OMD) or butaprost (Buta) on the Rho-associated coiled-coil-containing protein kinase (ROCK) inhibitor, ripasudil (Rip) on adipose tissue, two- or three-dimension (2D or 3D) cultures of 3T3-L1 cells were analyzed by lipid staining, the mRNA expression of adipogenesis-related genes, extracellular matrix (ECM) molecules including collagen (Col) -1, -4 and -6, and fibronectin (Fn), and the sizes and physical properties of 3D organoids, as measured by a micro-squeezer. The results indicate that adipogenesis induced (1) an enlargement of the 3D organoids; (2) a substantial enhancement in lipid staining as well as the expression of the *Ppar*γ, *Ap2* and *Leptin* genes; (3) a significant softening of the 3D organoids, the effects of which were all enhanced by Rip except for *Ppar*γ expression; and (4) a significant downregulation in *Col1* and *Fn*, and a significant upregulation in *Col4*, *Col6*, the effects of which were unchanged by Rip. When adding the EP2 agonist to Rip, (1) the sizes of the 3D organoids were reduced substantially; (2) lipid staining was increased (OMD), or decreased (Buta); (3) the stiffness of the 3D organoids was substantially increased in Buta; (4-1) the expression of *Ppar*γ was suppressed (2D, OMD) or increased (2D, Buta), and the expressions of *Ap2* were downregulated (2D, 3D) and *Leptin* was increased (2D) or decreased (3D), (4-2) all the expressions of four ECM molecules were upregulated in 2D (2D), and in 3D, the expression of *Col1*, *Col4* was upregulated. The collective findings reported herein indicate that the addition of an EP2 agonist, OMD or Buta significantly but differently modulate the Rip-induced effects on adipogenesis and the physical properties of 2D and 3D cultured 3T3-L1 cells.

## 1. Introduction

It has been reported that adipogenesis occurs in the determination phase; the conversion of mesenchymal stem cells (MSCs) to an adipocyte lineage or pre-adipocytes, and pre-adipocytes develop into mature adipocytes following the terminal differentiation phase [1]. During the terminal differentiation phase, several key transcription factors, including the peroxisome proliferator-activated receptor γ (*PPARγ*), the nuclear receptor, and the CCAAT-enhancer-binding protein (C/EBP) transcription factors are sequentially activated. Among these, *PPARγ*, a member of the nuclear-receptor superfamily, functions as the master regulatory gene for adipogenesis. Thus, *PPARγ* is not only required, but also sufficient, for adipogenesis as well as for the maintenance of adipocyte naturation [2,3,4,5]. It is also known that *PPARγ* and C/EBPα initiate the expression of various metabolic genes that are required for the maintenance of adipocyte phenotypes, including the fatty acid-binding protein 4 (FABP4; *AP2*) and glucose transporter 4 (GLUT4; SLC2A4), among others [1]. In addition, the expression of both *PPARγ* and C/EBPα are initiated by early transcription factors, C/EBPβ and C/EBPδ, which are activated within hours after stimulation for adipogenic differentiation [6].

Rho-associated coiled-coil-containing protein kinases (ROCKs) are recognized as important regulators of actin cytoskeleton remodeling in a variety of cells [7,8]. ROCK isoforms ROCK1 and ROCK2 share a high degree of homology within the amino and carboxyl termini, which make up the catalytic kinase domain and the Rho-binding domain (RBD), respectively, although their coiled-coil regions have only a 55% identity. Functionally, ROCKs are also known to play pivotal roles in cytokinesis, differentiation, apoptosis, glucose metabolism, cell adhesion/motility, and inflammation, in addition to the regulation of actin cytoskeleton remodeling [1,9,10,11]. Concerning the role of ROCKs in adipogenesis, it was reported that ROCK signaling inhibits adipocyte differentiation. In fact, in 3T3-L1 cells, it is known that ROCK2, but not ROCK1, is responsible for the suppression of adipogenesis, since ROCK inhibitors (ROCK-is), such as Y-27632 and fasudil, promote adipocyte differentiation [12]. Furthermore, by knockdown and genetic approaches, it was demonstrated that only ROCK2 induces anti-adipogenic activities within 3T3-L1 cells as well as mouse embryonic fibroblasts (MEFs) [13]. In our recent study, we reported the effects of pan-ROCK-is, ripasudil (Rip) and Y27632 on adipogenesis in two- or three-dimension (2D or 3D) cultures of 3T3-L1 cells and found that adipogenesis induced an increase in the sizes of the 3D organoids, in lipid staining and in the expression of mRNA of adipogenesis-related genes, and *Col4* and *Col6* were further enhanced by ROCK-is [14]. In addition, a micro-squeezer analysis demonstrated that the adipogenesis-induced softening of the 3D organoids was also further enhanced by ROCK-is. Based upon these collective findings, we concluded that ROCK-is significantly enhanced the production of large lipid-enriched 3T3-L1 3D organoids.

Alternatively, similar effects of ROCK-is on 2D and 3D cultured 3T3-L1 cells, as above, were also found for prostanoid EP2 agonists, omidenepag (OMD) and butaprost (Buta) [15]. This means that EP2 agonists also caused an increase in size and a decreased stiffness of the 3D 3T3-L1 organoids. However, it is known that EP2 receptors are also expressed in 3T3-L1 cells and that they are involved in different intracellular signaling pathways, in which the EP2 receptor is coupled to Gαs, resulting in an increase in cAMP [16] levels, as compared to those of ROCKs. Interestingly, it is known that both ROCK-is and EP2 agonists function to decrease intraocular pressures (IOPs) in several animal models [17,18], and in fact, among these, ROCK-i, Rip, and the EP2 agonist, OMD, are currently available for treating patients with glaucoma or ocular hypertension [19,20]. In addition, due to the local adverse effects of anti-glaucoma medications, significant attention has been paid to the issue of prostaglandin (PG)-induced peri-orbitopathy, such as the deepening of the upper eyelid sulcus (DUES) [21,22]. Recently, to study these anti-glaucoma medications for use in treating periocular tissues, especially orbital fatty tissues, the 3T3-L1 cell, a most frequently used preadipocyte cell line for adipogenesis related studies, has also been used [23]. Therefore, taking these similarities between ROCK-is and EP2 agonists toward the adipogenesis of the 3T3-L1 cells into account, it would be of great interest to learn more regarding the simultaneous effects of these materials, and this may rationally become the fundamental basis for estimating what would happen in cases where these drugs are clinically used together. In fact, several fixed combinations of anti-glaucoma medications are currently available in our glaucoma clinic [24]. 

Therefore, in the current study, we evaluated the effects of adding EP2 agonists (OMD and Buta) to pan-ROCK-i, Rip on adipogenesis of 3D 3T3-L1 organoids based on organoid volume, lipid production, ECM expression, and physical stiffness during the adipogenesis process. In terms of the rationale for using mouse preadipocytes, 3T3-L1 cells that were used in the present study, this cell line is the most extensively used line for adipogenesis-related research. As a result, the obtained observations should be useful for future studies in which human orbital fibroblasts and other cells are used.

## 2. Results

Our previous studies demonstrated that the EP2 agonist, OMD [15] and the ROCK inhibitor, Rip [14], had quite similar effects on the physical properties of the 3D 3T3-L1 organoids, although both drugs not only affect different signal transduction pathway, but also are differently regulated. To study this further, the additive effect of EP2-ag to the ROCK inhibitor, Rip on 2D and 3D cultured 3T3-L1 cells, their adipogenesis as well as ECM expression (2D and 3D) and the physical properties (3D) were examined. Since the 3D 3T3-L1 sphenoid maturation was complete within a 7-day culture period [14,15,25], all experiments described below involved the use of a 7-day culture 2D and 3D 3T3-L1 cells.

**Figure 1:**
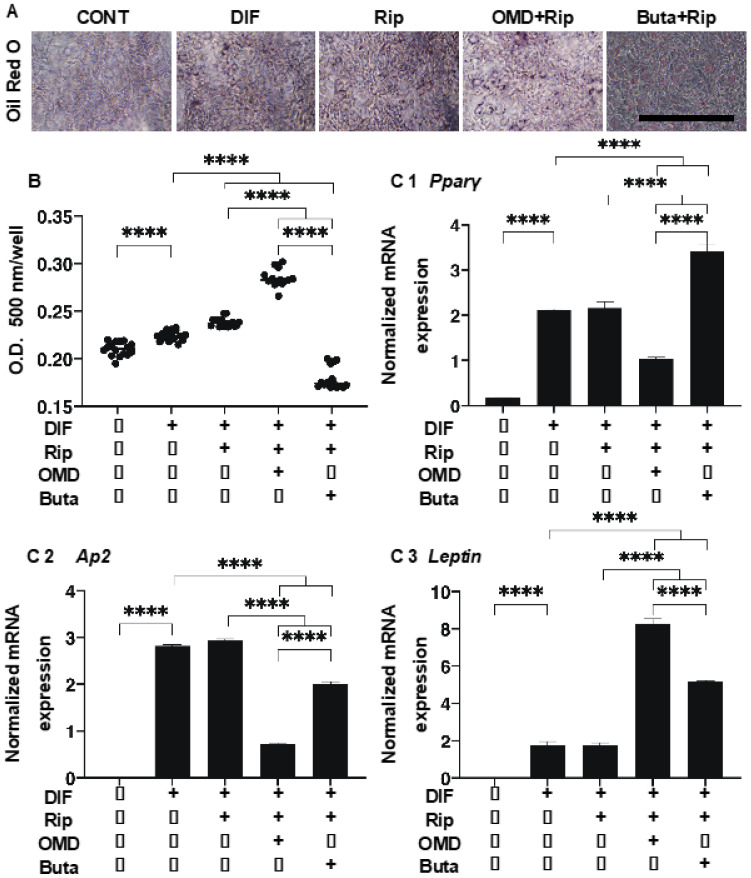
Additive effects of EP2 agonists to ripasudil (Rip) on the adipogenesis of 2D cultured 3T3-L1 cells. The 2D cultures of 3T3-L1 cells were prepared under several sets of conditions: preadipocytes of 3T3-L1 cells (DIF-) or their adipogenic differentiation (DIF+) with or without combinations of 10 μM ripasudil (Rip) and EP2 agonist, 100 nM omidenepag (OMD) or 100 nM butaprost (Buta). These specimens were subjected to analysis by Oil Red O lipid staining (panel **A**: representative phase contrast images, scale bar: 100 μm; and panel **B**: their staining intensities, O.D.) and qPCR of the master adipogenesis gene, *Pparγ*, *Ap2* and *Leptin* (panel **C** 1–3). All experiments were performed in triplicate using fresh preparations, each of which consisted of 5 specimens. Data are presented as the arithmetic mean ± the standard error of the mean (SEM). **** *p* < 0.001 (ANOVA followed by a Tukey’s multiple comparison test).

**Figure 2:**
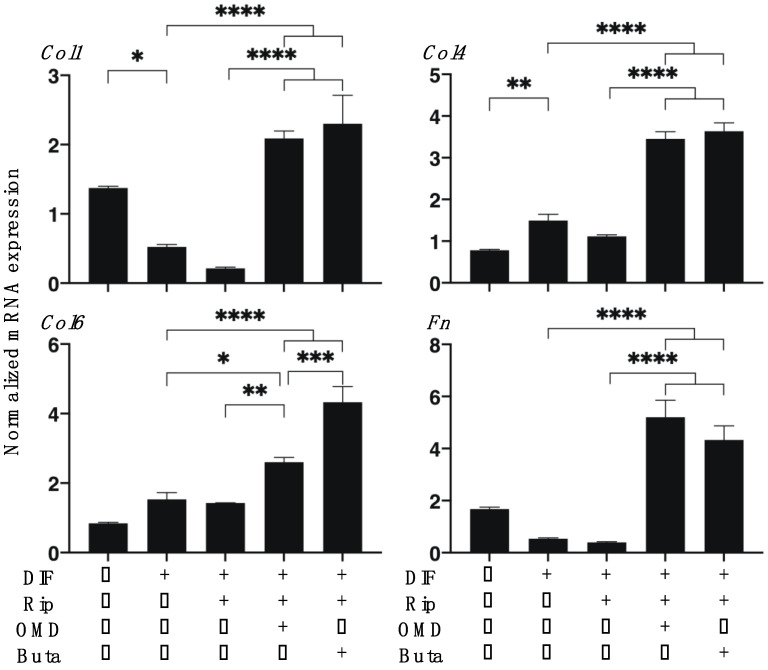
Additive effects of EP2 agonists to ripasudil (Rip) on the mRNA expression of ECMs of 2D cultured 3T3-L1 cells. The 2D cultures of 3T3-L1 cells were prepared under several sets of conditions: preadipocytes of 3T3-L1 cells (DIF-) or adipogenic differentiation (DIF+) with or without a combination of 10 μM ripasudil (Rip) and the EP2 agonist, 100 nM omidenepag (OMD) or 100 nM butaprost (Buta). These specimens were subjected to a qPCR analysis to estimate the expression of the mRNA of the major ECMs (*Col1*: collagen 1; *Col4*: collagen 4; *Col6*: collagen 6; *Fn*: fibronectin). All experiments were performed in triplicate using fresh preparations, and each experiment consisted of 5 samples. Data are presented as the arithmetic mean ± the standard error of the mean (SEM). * *p* < 0.05, ** *p* < 0.01, *** *p* < 0.005, **** *p* < 0.001 (ANOVA followed by a Tukey’s multiple comparison test).

As shown in Figure 1, lipid staining with Oil Red O, and the quantitative PCR of adipogenesis-related genes of the 2D cultured 3T3-L1 cells were significantly enhanced upon adipogenesis (DIF+) and these DIF+-induced effects were not significantly changed in the presence of Rip, as described in our previous study [14]. However, insufficient adipogenesis, as determined by Oil Red O, was also recognized in our previous studies [14,15,25]. This may be ascribed to differences in the efficacy of adipogenic differentiation between 2D and 3D cultures [14,15,25]. The addition of OMD to Rip resulted in a significant increase in staining intensities by Oil Red O and *Leptin* expression, and a significant decrease in *Ap2* expression was observed, while in contrast, the addition of Buta to Rip resulted in a significant increase in the expression of *Pparγ* and *Leptin*, whereas Oil Red O staining intensities and *Ap2* expression were substantially decreased. These results suggest that the addition of OMD or Buta to Rip have different effects on the adipogenesis processes of the 2D cultured 3T3-L1 cells. Upon DIF+, the mRNA expressions of ECMs in the 2D cultured 3T3-L1 cells, the levels of *Col1* and *Fn* were downregulated and the levels of *Col4* and *COL6* were upregulated. In the presence of Rip, the DIF+ induced changes in these ECMs were not significantly altered. In the case of the addition of EP2-ags to Rip, all four of these ECMs were substantially upregulated (Figure 2). 

**Figure 3:**
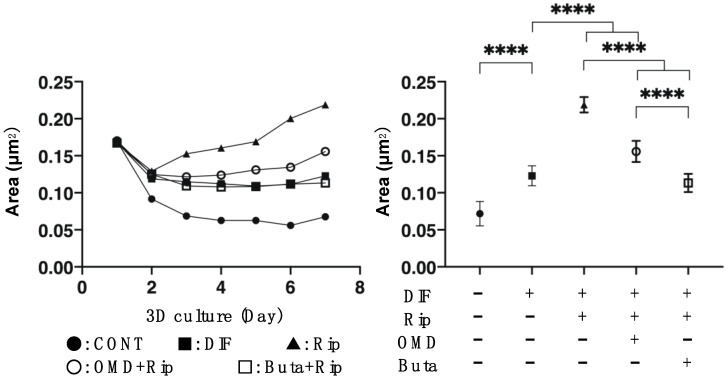
Additive effects of EP2 agonists to ripasudil (Rip) on the mean sizes of the 3T3-L1 3D organoids during adipogenesis. The 3D organoids of 3T3-L1 cells were prepared under several sets of conditions: preadipocytes of 3T3-L1 cells (DIF-) or adipogenic differentiation (DIF+) with or without a combination of 10 μM ripasudil (Rip) and the EP2 agonist, 100 nM omidenepag (OMD) or 100 nM butaprost (Buta). Their mean area sizes (μm^2^) were measured and plotted during a 7-day culture period (left panel) and those at Day 7 were compared among the experimental groups (right panel). All experiments were performed in triplicate using fresh preparations, each consisting of 16 organoids. Data are presented as the arithmetic mean ± the standard error of the mean (SEM). **** *p* < 0.001 (ANOVA followed by a Tukey’s multiple comparison test).

**Figure 4:**
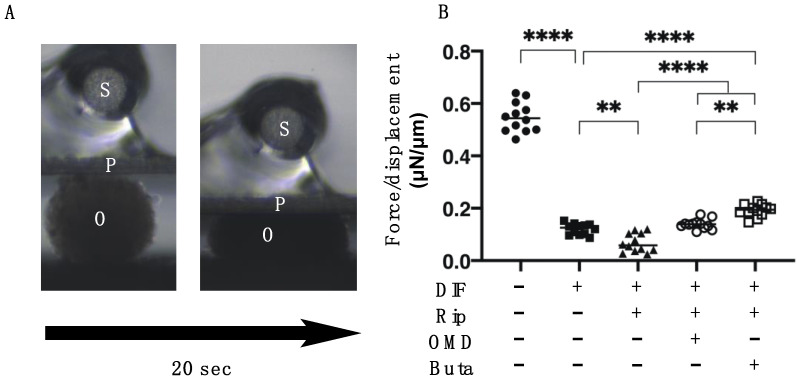
Additive effects of EP2 agonists to ripasudil (Rip) on the physical stiffness of 3T3-L1 3D organoids. The 3D organoids of 3T3-L1 cells were prepared under several sets of conditions: preadipocytes of 3T3-L1 cells (DIF-) or adipogenic differentiation (DIF+) with or without a combination of 10 μM ripasudil (Rip) and the EP2 agonist, 100 nM omidenepag (OMD) or 100 nM butaprost (Buta). The specimens collected on Day 7 were subjected to a physical solidity analysis. A single 3D organoid was placed on a 3 mm × 3 mm plate and was then compressed to 50% deformation during a period of 20 s, while being continuously monitored by a microscopic camera (panel **A**,**B**: S: micro-sensor of the mechanical force (μN), P: compression plate, O: single 3D organoid). Among above experimental conditions, the required force (μN) was measured and force/displacement (μN/μm) was potted (right panel). ** *p* < 0.01, **** *p* < 0.001 (ANOVA followed by Tukey’s multiple comparison test).

We then examined the additive effect of EP2-ags to Rip on the physical properties, size and stiffness, of 3D 3T3-L1 organoids. As shown Figure 3 and Figure 4, consistently with our previous study: (1) the sizes of the DIF- 3D 3T3-L1 organoids became smaller during the 6-day culture period; (2) upon adipogenesis (DIF+) their mean area sizes became significantly larger and softening was observed; and (3) such DIF+-induced effects were significantly enhanced in the presence of 10 μM Rip [14]. The addition of 100 nM EP2-ags diminished the Rip-induced enlargement and softening effects of the 3D organoids. 

**Figure 5:**
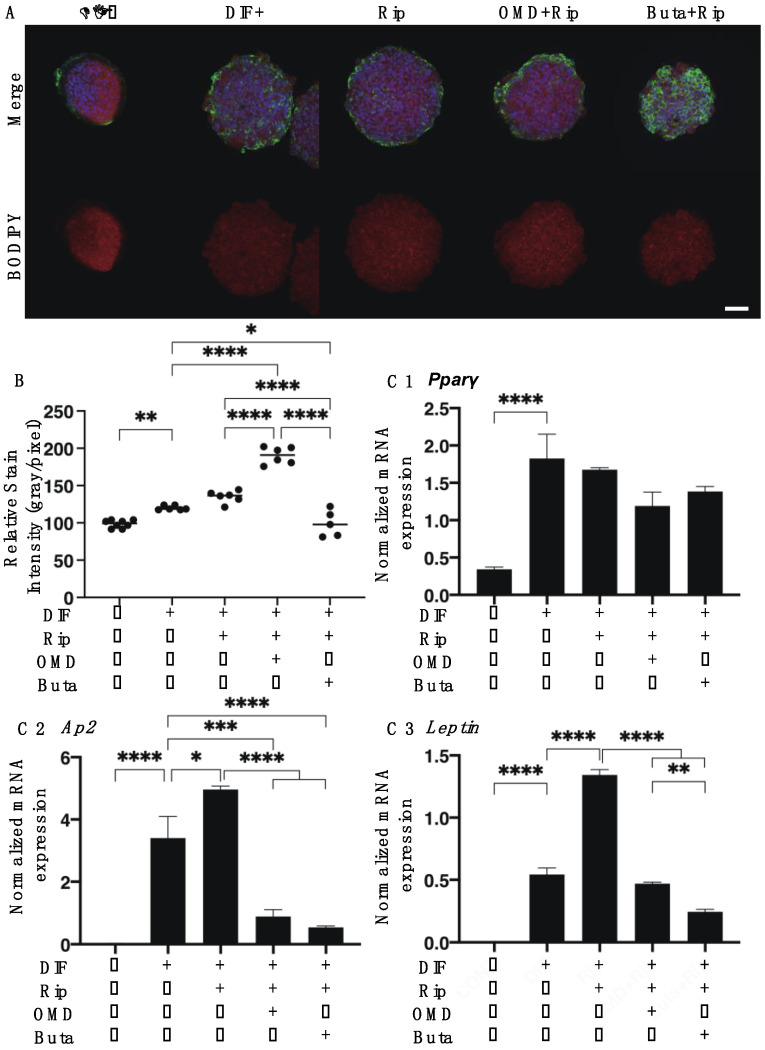
Additive effects of EP2 agonists to ripasudil (Rip) on the adipogenesis of 3T3-L1 3D organoids. The 3D organoids of 3T3-L1 cells were prepared under several sets of conditions: preadipocytes of 3T3-L1 cells (DIF-) or adipogenic differentiation (DIF+) with or without a combination of 10 μM ripasudil (Rip) and the EP2 agonist, 100 nM omidenepag (OMD) or 100 nM butaprost (Buta). These samples were immunostained with DAPI (blue), phalloidin (green) and BODIPY (red). Merge images and BODIPY images are shown in panel **A** (scale bar: 100 μm) and their staining intensities (gray/pixel) were plotted (panel **B**). The expression of the mRNA of adipogenesis-related genes including *Pparγ*, *Ap2* and *Leptin* under the above conditions were plotted and the data are shown in panel **C** 1–3. All experiments were performed in duplicate using fresh preparations, each consisting of 16 organoids. Data are presented as the arithmetic mean ± the standard error of the mean (SEM). * *p* < 0.05, ** *p* < 0.01, *** *p* < 0.005, **** *p* < 0.001 (ANOVA followed by a Tukey’s multiple comparison test).

**Figure 6:**
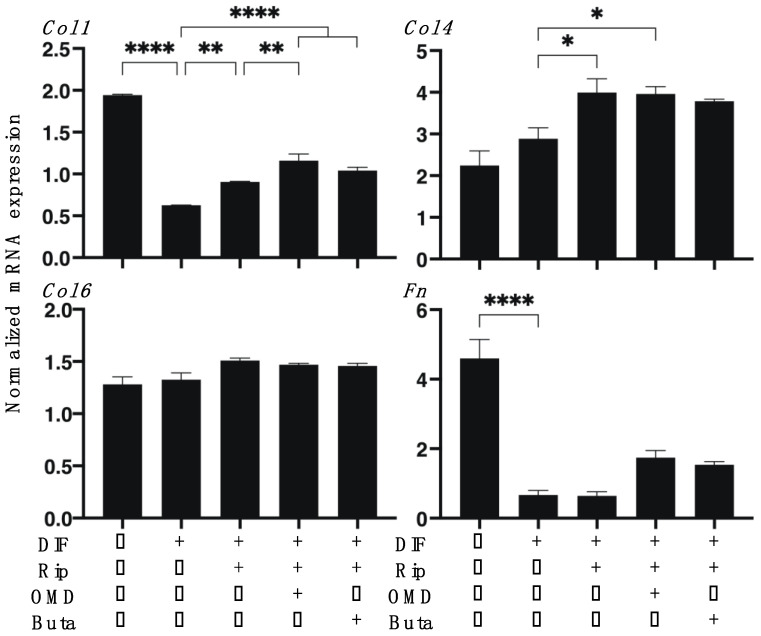
Additive effects of EP2 agonists to ripasudil (Rip) on the mRNA expression of ECMs in 3T3-L1 3D organoids. The 3D organoids of 3T3-L1 cells were prepared under several sets of conditions: preadipocytes of 3T3-L1 cells (DIF-) or adipogenic differentiation (DIF+) with or without the combination of 10 μM ripasudil (Rip) and the EP2 agonist, 100 nM omidenepag (OMD) or 100 nM butaprost (Buta). The specimens collected at Day 7 were subjected to a qPCR analysis to estimate the expression of mRNA for the ECMs (*Col1*: collagen 1; *Col4*: collagen 4; *Col6*: collagen 6; *Fn*: fibronectin). All experiments were performed in duplicate using fresh preparations, each of which consisted of 16 organoids. Data are presented as the arithmetic mean ± the standard error of the mean (SEM). * *p* < 0.05, ** *p* < 0.01, **** *p* < 0.001 (ANOVA followed by a Tukey’s multiple comparison test).

To study the additive effects of EP2-ags to Rip on adipogenesis in 3D 3T3-L1 organoids, lipid staining by BODIPY and the mRNA expression of adipogenesis-related genes including *Pparγ*, *Ap2* and *Leptin* were investigated. As shown in Figure 5A,B, in adipogenesis, the DIF+-induced enhancement in BODIPY staining intensities were not significantly altered in the presence of Rip, and these effects were further enhanced by the addition of Buta or suppressed by the addition of OMD. The mRNA expression of *Pparγ*, *Ap2* and *Leptin* in the 3D cultured 3T3-L1 cells were also significantly increased with adipogenesis (DIF+). The DIF+-induced effects on the expression of *Ap2* and *Leptin* were further enhanced by Rip, and this enhancement was substantially decreased by the addition of EP2-ags (Figure 5C). In terms of the expression of ECM molecules in the 3D 3T3-L1 organoids (Figure 6), the DIF+-induced downregulation of *Col1* and *Fn*, and the upregulation of *Col4* and *Col6* were observed, similar to the results of the 2D cell culture experiments described above. In the presence of Rip, the expressions of *Col1* and *Col4* were significant upregulated, and this upregulation of *Col1* was further enhanced by the addition of OMD to Rip (Table 1).

## 3. Discussion

It is well known that PGE2 is linked to four G protein-coupled receptor subtypes, EP1 through EP4 [26]. Functionally, EP1 increases intracellular Ca^2+^ levels but EP2 and EP4, or EP3 induce increases or decreases in cAMP levels, respectively [27]. Concerning these receptor distributions, EP2 is distributed within leukocytes, smooth muscle, the central nervous system (CNS), the reproductive system and ocular tissues, and functions in several signal transduction pathways [28]. An in vivo study demonstrated that EP2 receptor agonists induce a decrease in intraocular pressure (IOP) [29]. Among them, omidenepag isopropyl (OMDI), an OMD prodrug, which is metabolized into an active form, OMD, during its penetration into the eye, has recently been approved for the treatment of patients with glaucoma or ocular hypertension [22,29,30]. In terms of the effects of OMD on adipose tissue, it was reported that OMD had no effect on adipogenesis in 2D cultured 3T3-L1 cells [31]. However, in our subsequent study using the 2D and 3D cultures of the 3T3-L1 cells, we found that OMD induced a significant suppression of adipogenesis in these cultures [15], similar to PGE2 and PGF2α [32,33]. Furthermore, we also found that OMD significantly induced the enlargement and less stiffness in 3D 3T3-L1 organoid [15], while PGF2α resulted in substantially smaller and less stiff 3D 3T3-L1 organoids [25,34]. Therefore, these collective findings suggest that the EP2 agonist, OMD may exert some unknown effects on the architecture of 3D organoids, in addition to the suppression of adipogenesis. As another possibility, these drugs may have different efficacies toward *PPARγ* proteins which could cause different states of adipogenesis based upon following observations: (1) two isoform proteins of *PPARγ*, *PPARγ* 1 and *PPARγ* 2, in which *PPARγ* 1 lacks 30 N-terminal amino acids, which were found to be missing as compared with *PPARγ* 2 [35,36]; (2) *PPARγ* 1 is expressed in many tissues including adipose, skeletal muscle, heart, liver and large intestine despite the exclusive expression of *PPARγ* 2 within adipose tissues [37]; and (3) a previous study reported that that *PPARγ* 2 mainly activates adipogenesis in adipose tissue [38], although both *PPARγs* have an intrinsic adipogenesis-stimulating ability [39].

Similar to the EP2 agonist, OMD, a previous study demonstrated that ROCKs are negative regulators of adipocyte differentiation, and thus, ROCK-is, Y-27632 and fasudil, conversely promote adipocyte differentiation [12]. In addition, in our previous study, pan-ROCK-i, Rip was also found to significantly increase the adipogenesis of the 3D 3T3-L1 organoids, contrary to the EP2 agonist, OMD [14]. In addition, the effects of Rip and OMD on mRNA expressions of adipogenesis-related genes were also different (Table 1 in [15]). That is, Rip did not alter *Pparγ* but induced the upregulation of *Ap2* and *leptin* expressions, although an EP2 agonist, OMD caused the significant downregulation of *Pparγ* and *Ap2*. More interestingly, in the current study, the addition of OMD or Buta to Rip induced also different effects toward lipid staining (2D and 3D), that is, an increase or decrease, respectively. As for the additive effects of EP2 agonists to Rip on the mRNA expressions of adipogenesis-related genes, diverse effects of OMD vs. Buta as well as 2D vs. 3D were also recognized as follows; 2D; *Pparγ* (OMD; no significant change, Buta; downregulated), *Ap2* (OMD, Buta; no significant change), 3D; *Pparγ* (OMD, Buta; downregulated), *Ap2* (OMD, Buta; downregulated). In terms of these discrepancies of lipid staining and adipogenesis-related gene expressions among 2D and 3D cultures, it was not surprising because since the lipid staining intensities represent their spatial 3D distribution, those may not always correspond with their related gene expressions as observed in our previous study [25]. Taken together, these collective findings suggested that the addition of an EP2 agonist, OMD or Buta, significantly modulate the Rip-induced adipogenesis of the 3T3-L1 cells, and such additive effects were diverse between OMD and Buta.

It is known that the main adipocyte ECMs are COL1, 4, and 6, and FN and are modified during adipogenesis [40,41,42]. In fact, the expression of ECM is characteristically altered during in vivo and in vitro adipogenesis [43]. Previous studies using 2D or 3D cultures of 3T3-L1 preadipocytes revealed the in vitro remodeling from COL1- and FN-rich ECM in preadipocyte cells into further basal membrane type-rich ECMs, such as COL4 and 6, in adipocyte cells [40,41,42]. In the current study, such a downregulation of *Col1* and *Fn*, and the upregulation of *Col4* and *Col6* expression upon adipogenesis were also confirmed in both 2D and 3D cultures of the 3T3-L1 cells. As was observed in the effects of ROCK-i, Rip enhanced the adipogenesis-induced upregulation of *Col1* and *Col4* in 3D, but in 2D, while the expressions of *Col6* and *Fn* were not affected. While in contrast, as shown in our previous study (Table 1 in [15]), OMD or Buta induced the significant upregulation of *Col1* and downregulation of *Col4* and *Col6* in 3D [15]. Surprisingly, in the presence of both Rip and EP2-ags, ECM expressions were absolutely different, that is, all four were dramatically upregulated (Table 1 in [15]). Furthermore, lipid staining and adipogenesis-related gene expressions were also unexpectedly modulated as compared to the results by one of these drugs as above. 

Our previous studies revealed that despite similar effects towards the physical properties, sizes and stiffness, of the 3D 3T3-L1 organoids between Rip and EP2 agonists, their effects toward adipogenesis and ECM expressions were almost the opposite of each other [14,15]. In addition to the current unexpected simultaneous effects of both Rip and Ep2-ags toward 2D and 3D cultured 3T3-L1 cells, some unidentified mechanisms should be present. In fact, since it was suggested that as adverse effects, OMDI possesses a potential risk for cystoid macular edema and was especially advocated for patients undergoing cataract surgery with intraocular lens implantation or who are aphakic, the use of both Rip and OMDI may cause unexpected and unknown side effects. 

Thus, our current study facilitates better understanding the pharmacological aspects of Rip and OMDI as well as additional basic and clinical investigations to elucidate their simultaneous effects in human subjects. 

## 4. Materials and Methods

### 4.1. Adipocyte Cultures and the Differentiation of 3T3-L1 Cells

3T3-L1 preadipocytes (#EC86052701-G0, KAK) were grown in 2D cultures and subjected to further 3D cultures using a hanging droplet culture plate (# HDP1385, Sigma-Aldrich, St. Louis, MO, USA) during 7 days, as described previously [15,25,34]. In terms of this 7-day culture period, our previous study revealed that the 3D 3T3-L1 sphenoid maturation was complete and that the cultures were stabilized after a 7-day culture period [14]. Therefore, for the following experiments, these fresh 3D 3T3-L1 sphenoids were used as they were also used in our previous studies [14,15,25]. The adipogenic differentiation in the 2D or 3D cultured 3T3-L1 cells [15,25,34] was initiated by the addition of 250 nM dexamethasone, 10 nM T3 for two days and with 10 μM troglitazone, and 1 μg/mL insulin for the next three days. To study the drug efficacy of the pan-ROCK-i, Rip and EP2 agonists, 10 μM Rip without or with 100 µM OMD or Buta were added during the course of adipogenic induction. These drug concentrations were confirmed to be optimum for following analysis in our previous studies [14,15]. 

### 4.2. Oil Red O (2D) or BODIPY (3D) Lipid Staining

2D cultured 3T3-L1 cells, as described above, were subjected to Oil Red O staining, as described by the manufacturer protocol (Abcam, Cambridge, UK, #133102).

Regarding BODIPY lipid staining [15,25,34], 4% paraformaldehyde (PFA) fixed 3D organoids were incubated in 0.1% BODIPY (#D3922, Thermo Fisher Scientific, Waltham, MA, USA), 0.1% DAPI (#D523, Doujin, Tokyo, Japan) and 0.1% phalloidin (#20553, Funakoshi, Tokyo, Japan) in phosphate buffered saline containing 3% bovine serum albumin for 3 h. The microscopy images of above lipid stainings were taken by a Nikon A1 confocal microscope (Tokyo, Japan) and quantified using the Image J software version 2.0.0 (NIH, Bethesda, Rockville, MD, USA). 

### 4.3. Quantitative PCR

Total RNA extraction (a RNeasy mini kit, Qiagen, Valencia, CA, USA), reverse transcription (the SuperScript IV kit, Invitrogen, Carlsbad, CA, USA) and real-time PCR (Applied Biosystems/Thermo Fisher Scientific) were performed as described previously [15,25,34]. The normalization of the cDNA levels of the respective genes was performed by the standard curve method for relative quantitation using the 36B4 (*Rplp0*) gene as the housekeeping gene [44]. Sequences of the primers and Taqman probes used are shown in Table S1.

### 4.4. Micro-Indentation Force Analysis

The micro-indentation force for a single living 3D organoid was studied by a micro-squeezer (CellScale, Waterloo, ON, Canada) as described previously [15,25,34]. Briefly, the force (μN) required to achieve a 50% deformation during a period of 20 s (force/displacement, μN/μm) was measured.

### 4.5. Statistical Analysis

All statistical analyses were performed using the Graph Pad Prism 8 (GraphPad Software, San Diego, CA, USA). To analyze the difference between groups, a grouped analysis with two-way analysis of variance (ANOVA) followed by a Tukey’s multiple comparison test was performed. Data are presented as the arithmetic mean ± the standard error of the mean (SEM).

## Figures and Tables

**Table 1:** Summary of effects of single or combinations of Rip and EP2 agonists on the physical properties of the 3D 3T3-L1 organoids and adipogenesis and gene expressions of 2D and 3D cultured 3T3-L1 cells.

		Rip	OMD *	Buta *	Rip + OMD	Rip + Buta
size		↑↑↑	(−)	(−)	↑↑↑	(−)
stiffness		↓	(−)	(−)	(−)	↑↑↑
lipid stain	2D	(−)	↓↓↓	↓↓↓	↑↑↑	↓↓↓
	3D	(−)	↓	↓	↑↑↑	↓↓↓
*Pparγ*	2D	(−)	(−)	↓	↓↓↓	↑↑↑
	3D	(−)	↓↓↓	↓↓↓	(−)	(−)
*Ap2*	2D	(−)	(−)	(−)	↓↓↓	↓↓↓
	3D	↑	↓↓↓	↓↓↓	↓↓↓	↓↓↓
*Leptin*	2D	(−)	N.D.	N.D.	↑↑↑	↑↑↑
	3D	↑↑↑	N.D.	N.D.	(−)	(−)
*Col1*	2D	(−)	(−)	(−)	↑↑↑	↑↑↑
	3D	↑↑	↑↑↑	↑↑↑	↑↑↑	↑↑↑
*Col4*	2D	(−)	(−)	(−)	↑↑↑	↑↑↑
	3D	↑	↓↓↓	↓↓↓	↑	(−)
*Col6*	2D	(−)	(−)	(−)	↑	↑↑↑
	3D	(−)	↓↓	↓↓↓	(−)	(−)
*Fn*	2D	(−)	(−)	(−)	↑↑↑	↑↑↑
	3D	(−)	(−)	(−)	(−)	(−)

Rip: ripasudil; OMD: omidenepag; Buta: butaprost; 2D: two-dimension culture; 3D: three-dimension culture; *Pparγ*: peroxisome proliferator-pctivated peceptor γ; *Ap2*: adipocyte protein 2; *Col*: collagen; Fn: fibronectin; (−): not significant change; ↑: significant increase (*p* < 0.05); ↑↑: significant increase (*p* < 0.01); ↑↑↑: significant increase (*p* < 0.01); ↓: significant decrease (*p* < 0.05); ↓↓: significant decrease (*p* < 0.01); ↓↓↓: significant decrease (*p* < 0.005); N.D.: not determined; * results are recruited from our previous studies [15].

## Data Availability

The data that support the findings of this study are available from the corresponding author upon reasonable request.

## Supplementary Materials

The following are available online at https://www.mdpi.com/article/10.3390/ijms22094648/s1.
